# “Tour de France” data for the improvement of energy consumption in devices powered by limited energy sources

**DOI:** 10.1016/j.dib.2020.106334

**Published:** 2020-09-28

**Authors:** Hamzaoui Khalil Ibrahim, Dahmani Soufiane, Boulet Pierre

**Affiliations:** a Univ. Lille, CNRS, Centrale Lille, UMR 9189 - CRIStAL - Centre de Recherche en Informatique Signal et Automatique de Lille, Lille F-59000, France; b Univ. Mohammed Premier, FSO, Lab.LANOL, Oujda 60000, Morocco

**Keywords:** Energy consumption, Collection of data, Internet of things, Data processing

## Abstract

In this article, we propose a dataset about the energy consumption of mobile devices that was collected as part of a “Tour de France” with an electrical wheelchair. Part of these data was used to propose a mathematical model based on an experimental methodology of the energy consumed in mobile devices.

Based on these precise measurements in a real environment, we have elaborated predictive models of energy consumption. The objective of this paper is to make accessible the data related to these publications to other researchers in several fields of science (computer science, telecommunications, meteorological science, artificial intelligence, statistics...).

To our knowledge, this is the first publication of a dataset recording real world energy consumption data in mobile devices.

## Specifications Table

**Table:** 

Subject	Computer Science, Computational Theory and Mathematics
More specific subject area	Improvement of energy consumption in devices powered by limited energy sources
Type of data	Text (csv file)
How data was acquired collection	These experimental data were collected during a 33 day long “Tour de France” with an electrical wheelchair: https://www.univ-lille.fr/fileadmin/user_upload/presse/20160427-DP-1er_Tour_de_France_en_fauteuil_electrique.pdf The data were collected on the basis of several scenarios in different places (3006 kilometers(Km)). These measures were operated out in a real environment.
Parameters for data	Tablet HP Pro Slate 8 (Snap Dragon), Trepn Profiler V6.2s [1], Cpu Frequency, Cronoid 3.5.1 [2]
Data format	Raw
Description of data	Scientific data was collected on the basis of several collection scenarios in different places. These measures were operated out in a real environment and confronted with the consumption measures carried out beforehand in a controlled environment. After each experiment, the obtained data is stored in a csv file with a large number of parameters and data retrieved.
Data source location	Cities : Lille, Amiens, Rouen, Le Havre, Caen, Rennes, Nantes, La Rochelles, Bordeaux, Toulouse, Montpellier, Avignon, Grenoble, Lyon, Dijon, Troyes, Paris, Valenciennes. Country: France.
Data accessibility	The raw data are available at: https://zenodo.org/record/3739472, with a Creative Commons Attribution 4.0 International license
Repository name	Zenodo : “Tour de France” data for the improvement of energy consumption in devices powered by limited energy sources.
Related research article	Author’s name: Khalil Ibrahim HAMZAOUI, Mohammed BERRAJAA, Mostafa AZIZI, Giuseppe LIPARI, Pierre BOULET Title: Measurement-based methodology for modelling the energy consumption of mobile devices Journal: International Journal of Reasoning based Intelligent Systems DOI: https://doi.org/10.1504/IJRIS.2020.105007[3]

## Value of the Data

• The presented experimental data are useful to measure several metrics related to the energy variation in a mobile device.
• The data are beneficial for all the scientists who are exploring energy consumption in devices powered by limited energy sources as a knowledge resource.
• The data can be used to analyze the load of the Central Processing Unit (CPU) and the Graphics Processing Unit (GPU) as well as to make a more detailed follow-up of the energy variation on other type of mobile environment.
• The provided data can be used for further analysis in order to define models to improve energy handling in mobile devices, to define other types of scenarios given the large size of the data measured during the collection, or to optimize our methodology (based on part of the dataset [3], [4]).

## 1 Data Description

The presented research is part of the development of mathematical models, for modeling and evaluating the energy cost in mobile environments.

Our study focused on the Android system (version 4.4.4 (KitKat)). The main objective of the data collection was to model the energy consumption of a particular application running on a mobile device.

The data collection made possible to develop a specific mathematical model of energy consumption for monitoring the energy consumption of playing a local video, remote video streaming as well as in the case of navigating with a Global Positioning System (GPS) by acting on the following parameters:

• Processor frequency,
• Initial battery level,
• Dissipated energy.

This analyse is focused on the development and measurement of energy costs. Our work focused on monitoring energy consumption based on several experimental scenarios.

Scientific data was collected on the basis of several scenarios in different locations. These measures were carried out in a real environment and confronted with the consumption measures made beforehand in a controlled environment.

Collecting and analyzing massive data in a real environment and diversifying it in a reasonable time creates a scientific challenge. The objective of the carried out research is to model the energy consumption of a particular application running on a mobile device.

In the rest of our paper, we will base ourselves on two types of case studies:

• The disconnected mode in which all the measurements were carried out in the presence of only Global System for Mobile Communications (GSM).
• Connected mode where all connection techniques have been activated (3G, 4G, Wi-Fi, GPS, etc.) depending on the chosen scenario.

The raw data are stored in text files (in CSV format). They are divided into four main groups depending on the chosen scenarios. Table 1 presents the different characteristics of the group of scenarios used for the evaluation.

**Table 1:** Characteristics of the reference scenarios.

Model name	Meaning	Mode	Description
LVFF	Local Video	Disconnected	Monitoring the energy
	with fixed		behavior of a local video
	frequency		under a fixed frequency
LVVF	Local Video	Disconnected	Monitoring of energy
	with variable		behavior by of a local
	frequency		video at a variable
			frequency
NFF	Navigation	Connected	Monitoring of the energy
	with fixed		behavior by launching
	frequency		navigation via GPS under
			a fixed frequency
NVF	Navigation	Connected	Monitoring of the energy
	with variable		behavior by launching
	frequency		navigation via GPS
			at a variable frequency

The files are named as follows:

**scenario_name_freq_GHz_[Option]_[departure_city]_[arrival_city]**

Table 2 shows some examples of measurement files for all scenarios in disconnected mode. The list of all files relating to the disconnected mode is presented in Tables 3 and  4.

**Table 2:** Description of the disconnected scenario file names.

File name Scenario	Meaning
LVFF_freq0.3GHz	Measurement with the LVFF scenario by setting the
_Caen_Domfront.csv	frequency at 0.3GHz between Cean (departure city)
	and Domfront (arrival city).
LVFF_freq0.3GHz	Information about present applications during the
_Caen_Domfront_info.csv	experiment, Percentage of use of CPU resources,
	Average of use of virtual memory per application,
	Average use of real memory per application,
	Maximum use of real memory per application,
	statistics of the active sensors between
	Caen and Domfront.
LVVF_default_freq	Mesurement with LVVF scenario with variable
_Lille_Amiens.csv	frequency (the default frequency) between
	Lille and Amiens.
LVVF_default_freq	Information about present applications during the
_Lille_Amiens_info.csv	experiment, Percentage of use of CPU resources,
	Average of use of virtual memory per application,
	Average use of real memory per application,
	Maximum use of real memory per application,
	statistics of the active sensors between
	Lille and Amiens.

**Table 3:** List of the LVFF files in the dataset.

File name	Number of lines
LVFF_freq0.3GHz_Caen_Domfront.csv	25,603
LVFF_freq0.3GHz_Caen_Domfront_info.csv	0
LVFF_freq0.3GHz_Castelsarrasin_Toulouse.csv	60,723
LVFF_freq0.3GHz_Castelsarrasin_Toulouse_info.csv	124,039
LVFF_freq0.3GHz_Domfront_Rennes.csv	52,810
LVFF_freq0.3GHz_Domfront_Rennes_info.csv	87,105
LVFF_freq0.3GHz_Rouen_Le_Havre2.csv	17,675
LVFF_freq0.3GHz_Rouen_Le_Havre2_info.csv	28,114
LVFF_freq1.0GHz_Le_Havre_Domfront.csv	27,000
LVFF_freq1.0GHz_Le_Havre_Domfront_info.csv	0
LVFF_freq1.5GHz_Beziers_Montpellier.csv	60,155
LVFF_freq1.5GHz_Beziers_Montpellier_info.csv	123,842
LVFF_freq1.5GHz_Carcan_Bordeaux.csv	67,142
LVFF_freq1.5GHz_Carcan_Bordeaux_info.csv	128,839
LVFF_freq1.5GHz_Le_Havre_Domfront.csv	27,000
LVFF_freq1.5GHz_Le_Havre_Domfront_info.csv	0
LVFF_freq1.7GHz_Carcans_Bordeaux.csv	39,826
LVFF_freq1.7GHz_Carcans_Bordeaux_info.csv	76,807
LVFF_freq2.2GHz_Bordeaux_Marmande1.csv	65,083
LVFF_freq2.2GHz_Bordeaux_Marmande1_info.csv	150,503
LVFF_freq2.2GHz_Bordeaux_Marmande2.csv	57,728
LVFF_freq2.2GHz_Bordeaux_Marmande2_info.csv	146,489
LVFF_freq2.2GHz_Rouen_Le_Havre1.csv	47,520
LVFF_freq2.2GHz_Rouen_Le_Havre1_info.csv	114,434
LVFF_freq2.2GHz_Valence_Grenoble.csv	59,191
LVFF_freq2.2GHz_Valence_Grenoble_info.csv	137,361

**Table 4:** list of the LVVF files in the dataset.

File name	Number of lines
LVVF_default_freq_Chateau_Thierry_Reims.csv	53,110
LVVF_default_freq_Chateau_Thierry_Reims_info.csv	113,303
LVVF_default_freq_Lille_Amiens.csv	52,227
LVVF_default_freq_Lille_Amiens_info.csv	103,631
LVVF_default_freq_Narbonne_Beziers.csv	15,874
LVVF_default_freq_Narbonne_Beziers_info.csv	28,268

For convenience, each measurement file is divided into two sub-files: The first one contains the list of measurements made during the experiment. The second one has the same name with the suffix “info” which gives information about active applications during the experiment.

The empty files “info files” (Zero line), means that the option which gives information was deactivated at the time of collection for these experiments.

The connected mode can be described as follows in Table 5

**Table 5:** Description of the connected scenario file names.

File name Scenario	Meaning
NFF_freq1.0GHz_GPS	Mesurement with NVF scenario with
_facebook_Provins_Paris.csv	variable frequency (the default frequency)
	between Provins and Paris.
NFF_freq1.0GHz_GPS	Information about present applications during
_facebook_Provins_Paris_info.csv	the experiment, Percentage of use of CPU
	resources, Average of use of virtual memory
	per application, Average use of real memory
	per application, Maximum use of real memory
	per application, statistics of the active sensors
	between Provins and Paris.
NVF_default_freq_GPS	Mesurement with NVF scenario with variable
_facebook _Provins_Paris.csv	frequency (the default frequency) between
	Provins and Paris.
NVF_default_freq_GPS	Information about present applications during the
_facebook_Provins_Paris_info.csv	experiment, Percentage of use of CPU resources,
	Average of use of virtual memory per application,
	Average use of real memory per application,
	Maximum use of real memory per application,
	statistics of the active sensors between
	Provins and Paris.
NVF_Optimal_Freq_GPS	Mesurement with NVF scenario with variable
_facebook_Lyon_Tournus.csv	frequency, choosed by tool of mesurement
	(Optimal frequency) between Lyon and Tornus.
NVF_Optimal_Freq_GPS	Information about present applications during
_facebook_Lyon_Tournus_info.csv	the experiment, Percentage of use of CPU resources,
	Average of use of virtual memory per application,
	Average use of real memory per application,
	Maximum use of real memory per application,
	statistics of the active sensors between
	Lyon and Tournus.

Tables 6 and 7 show the storage structure of the connected mode files.

**Table 6:** composition of NFF files in the dataset.

File name	Number of lines
NFF_freq0.3GHz_GPS_facebook_Nantes_La_Rochelle.csv	166,978
NFF_freq0.3GHz_GPS_facebook_Nantes_La_Rochelle_info.csv	1,048,576
NFF_freq0.3GHz_GPS_facebook_Paris_Chateau_Thierry.csv	50,198
NFF_freq0.3GHz_GPS_facebook_Paris_Chateau_Thierry_info.csv	33,904
NFF_freq0.652GHz_facebook_La_Rochelle_Royan.csv	76,726
NFF_freq0.652GHz_facebook_La_Rochelle_Royan_info.csv	50,716
NFF_freq0.652GHz_GPS_facebook_Avignon_Orange.csv	75,297
NFF_freq0.652GHz_GPS_facebook_Avignon_Orange_info.csv	49,298
NFF_freq0.960GHz_GPS_Tonneins_Castelsarrasin.csv	165,784
NFF_freq0.960GHz_GPS_Tonneins_Castelsarrasin_info.csv	105,922
NFF_freq1.0GHz_GPS_facebook_Dijon_Provins.csv	88,883
NFF_freq1.0GHz_GPS_facebook_Dijon_Provins_info.csv	60,762
NFF_freq1.0GHz_GPS_facebook_Provins_Paris.csv	92,149
NFF_freq1.0GHz_GPS_facebook_Provins_Paris_info.csv	61,302
NFF_freq1.0GHz_GPS_google_maps_Royan_Carcans.csv	183,642
NFF_freq1.0GHz_GPS_google_maps_Royan_Carcans_info.csv	124,906
NFF_freq1.5GHz_GPS_facebook_Troyes_Provins.csv	104,792
NFF_freq1.5GHz_GPS_facebook_Troyes_Provins_info.csv	69,203
NFF_freq1.5GHz_GPS_facebook_Viviers_Romains_sur_Isere.csv	135,330
NFF_freq1.5GHz_GPS_facebook_Viviers_Romains_sur_Isere_info.csv	93,228
NFF_freq1.96GHz_GPS_facebook_Tournus_Dijon.csv	140,454
NFF_freq1.96GHz_GPS_facebook_Tournus_Dijon_info.csv	96,312
NFF_freq2.2GHz_GPS_facebook_Rennes_Nantes.csv	36,848
NFF_freq2.2GHz_GPS_facebook_Rennes_Nantes_info.csv	26,773

**Table 7:** list of the NVF files in the dataset.

File name	Number of lines
NVF_default_freq_GPS_facebook_Avignon_Viviers.csv	167,713
NVF_default_freq_GPS_facebook_Avignon_Viviers_info.csv	116,713
NVF_default_freq_GPS_facebook_Dijon_Montbard.csv	163,712
NVF_default_freq_GPS_facebook_Dijon_Montbard_info.csv	113,853
NVF_default_freq_GPS_facebook_Laon_Valenciennes.csv	106,244
NVF_default_freq_GPS_facebook_Laon_Valenciennes_info.csv	74,877
NVF_default_freq_GPS_facebook_Lyon_Tournus.csv	161,025
NVF_default_freq_GPS_facebook_Lyon_Tournus_info.csv	113,831
NVF_default_freq_GPS_facebook_Nantes_La_Rochelle.csv	166,978
NVF_default_freq_GPS_facebook_Nantes_La_Rochelle_info.csv	124,942
NVF_default_freq_GPS_facebook_Paris_Chateau_Thierry.csv	136,027
NVF_default_freq_GPS_facebook_Paris_Chateau_Thierry_info.csv	94,401
NVF_default_freq_GPS_facebook_Provins_Paris.csv	174,955
NVF_default_freq_GPS_facebook_Provins_Paris_info.csv	118,494
NVF_default_freq_GPS_facebook_Rennes_Nantes.csv	195,970
NVF_default_freq_GPS_facebook_Rennes_Nantes_info.csv	132,585
NVF_default_freq_GPS_facebook_Tournus_Dijon.csv	66,718
NVF_default_freq_GPS_facebook_Tournus_Dijon_info.csv	45,905
NVF_default_freq_music_with_WiFi_Carcassonne_Narbonne.csv	188,758
NVF_default_freq_music_with_WiFi_Carcassonne_Narbonne_info.csv	131,415
NVF_default_freq_youtube_with_WiFi_Carcassonne_Narbonne.csv	52,069
NVF_default_freq_youtube_with_WiFi_Carcassonne_Narbonne_info.csv	32,870
NVF_Optimal_freq_GPS_facebook_Avignon_Viviers.csv	805
NVF_Optimal_freq_GPS_facebook_Avignon_Viviers_info.csv	563
NVF_Optimal_Freq_GPS_facebook_Lyon_Tournus.csv	720
NVF_Optimal_freq_GPS_facebook_Lyon_Tournus_info.csv	486

### 1.1 Data preparation

The measurement file is represented in Tables 8 and 9 for the experiment that was carried out on 6 May 2016. The obtained data are stored in

**Table 8:** Example of raw measured data (part 1).

Time	Cpu1	Cpu1	Cpu2	Cpu2	Cpu3	Cpu3	Cpu4	Cpu4
[ms]	Freq	Load	Freq	Load	Freq	Load	Freq	Load
	[kHz]	[%]	[kHz]	[%]	[kHz]	[%]	[kHz]	[%]
1,208,013	300,000	42	0	0	0	0	2,265,600	57
1,208,113	1,728,000	50	0	0	0	0	2,265,600	41
1,208,218	300,000	90	0	0	0	0	2,265,600	65
1,208,272	1,728,000	100	0	0	0	0	2,265,600	66
1,208,373	300,000	66	0	0	0	0	2,265,600	41
1,208,475	1,728,000	20	0	0	0	0	2,265,600	11
⋅⋅⋅	⋅⋅⋅	⋅⋅⋅	⋅⋅⋅	⋅⋅⋅	⋅⋅⋅	⋅⋅⋅	⋅⋅⋅	⋅⋅⋅
3778779	1728000	33	0	0	0	0	2265600	18

**Table 9:** Example of raw measured data (part 2).

Time	Memory	Screen	Battery	Battery	GPU	CPU	CPU
[ms]	Usage	Brigh-	Power	Remaining	Load	Load	Temp
	[kb]	tness	[*μ*W]	[%]	[%]	[%]	[1/10 C]
1,208,013	1,797,872	15	935,452	19	0	42	460
1,208,113	1,798,120	15	1,120,303	19	0	50	460
1,208,218	1,798,860	15	1,061,060	19	0	42	460
1,208,272	1,798,860	15	1,955,387	19	0	78	460
1,208,373	1,797,636	15	680,417	19	0	80	460
1,208,475	1,797,704	15	380,873	19	0	45	460
⋅⋅⋅	⋅⋅⋅	⋅⋅⋅	⋅⋅⋅	⋅⋅⋅	⋅⋅⋅	⋅⋅⋅	⋅⋅⋅
3,778,779	1,748,624	15	3,15,252	19	0	38	530

“LVFF_freq0.3GHz_Caen_Domfront.csv”. All our raw data are available at https://zenodo.org/record/3739472

#### Meaning of the columns of Table 8:

• Time [ms]: The interval of measurements in milliseconds (ms),
• Cpu1 Freq [kHz]; Cpu2 Freq [kHz]; Cpu3 Freq [kHz]; Cpu4Freq [kHz]: The respective frequencies of the 4 Central Processing Units (CPUs) in kiloHertz [kHz].
• Cpu1 Load, Cpu2 Load, Cpu3 Load, Cpu4 Load: Percentages of respective loads of the 4 CPUs [%].

#### Meaning of the columns of Table 9:

• Time [ms]: The interval of measurements in milliseconds (ms),
• Memory Usage [Kb]: Memory used per measurement interval in Kilobyte [Kb],
• Screen Brightness: Screen brightness state,
• Battery Power* [*μ*W]: Power consumption per measurement interval in microwatt(*μ*W),
• Battery Remaining [%]: Remaining battery capacity,
• GPU Load [%]: Total Graphics Processing Unit load,
• CPU Load [%]: Total CPU load.
• CPU Temp [1/10 C]: Processor Temperature per measurement interval (by 1/10^∘^C).

The average number of accesses per application and statistics on the sensors (Table 10), the “info file” contains also information about the percentage of use of CPU resources, the average use of virtual memory per application, the maximum use of virtual memory per application, average real memory usage per application and maximum real memory usage per application, all these information are detailed in the Table 11.

**Table 10:** Statistics and information about active applications.

Identifier of the	Type of	Average Number of
Application Sensor	Application	Accesses per Application.
200	Mobile Data State	0.00
205	Battery Remaining %	47.634
206	Battery Status	0.00
328	Memory Usage	1772465.436
331	Screen Brightness	247.383
332	Battery Power	989798.485
400	GPU Frequency	226874.220
401	GPU Load	32.800
600	CPU Load	75.213
1000	CPU1 Frequency	856941.408
1001	CPU2 Frequency	664897.739
1002	CPU3 Frequency	812255.094
1003	CPU4 Frequency	860962.047
1096	CPU1 Load	76.488
1097	CPU2 Load	68.417
1098	CPU3 Load	73.458
1099	CPU4 Load	74.583

**Table 11:** Example of system resource allocation.

Applications	CPU	Average	Max	Average	Max
	[%]	Virtual	Virtual	Resident	Resident
		Memory	Memory	Set Size	Set Size
		Size [MB]	Size [MB]	[MB]	[MB]
Facebook	0.18	1784.41	1794.03	26.99	34.0
GsmaService	0.0018	1483.33	1485.60	5.79	6.09
Clock	0.006	257.32	1493.86	1.42	8.37
CPU Frequency	5.51E-5	606.2	1489.42	2.79	7.64
Google Play Store	0.004	15.80	1545.80	8.33	9.59
Launcher3	0.004	1589.49	1589.49	12.08	13.789
Services Google Play	0.34	4096.29	6540.66	34.89	60.18
Google Partner	5.04E-4	24.55	1486.86	0.09	5.97
Configuration					
Play-Fi	0.0017	1483.88	1483.88	5.05	5.69
Personal Dictionary	0.007	99.57	1491.23	0.52	7.94
YouTube	20.51	1966.10	1985.42	42.51	48.83
Cron Tasker Free	0.06	1330.37	1492.48	7.004	8.63
Gmail	0.050	893.47	1834.24	12.28	33.15
Google Application	0.032	3084.76	3091.49	16.81	19.653
Cronoid	0.62	1507.74	1520.84	7.97	8.94
Power Battery	0.47	1682.2	3114.73	10.07	29.05
Hangouts	0.05	19.82	1665.26	0.18	21.22
Google+	0.031	64.793	1545.664	0.487	12.356
Google keyboard	0.078	1548.13	1607.44	11.58	14.71
Mobile Network	0.28	3034.45	3035.91	14.26	16.59
Configuration					
SmartcardService	0.006	2987.34	2991.18	13.007	14.622
System Interface	0.25	1727.72	1805.57	41.59	51.07
Messenger	0.14	1871.18	1910.15	46.07	56.89
SVI Settings	3.46	9172.51	9217.14	55.79	64.84
Multimedia Storage	0.002	277.25	1495.03	1.28	7.613

The meaning of the applications not recognized are:

• **GsmaService**: The GSMA provides a range of services to assist the broader mobile industry.
• **Launcher3**: Application launcher, it is the equivalent of the desktop on Android.
• **Google Partner Configuration**: Application that helps for run and configure applications in conjunction with Google products.
• **Play-Fi**: Application for set up to streaming music.
• **Cron Tasker Free**: Application for Scheduling tasks simply by configuring the time.
• **SmartcardService**: The smart card service is a included system in the operating system in a root directory. it appears to the smart card provider [5].
• **SVI Settings**: A Switch Virtual Interfaces Represents a logical Layer 3 interface on a switch.

## 2 Experimental Design, Materials and Methods

### 2.1 Methodology

This dataset was used to model the energy consumption of one or more specific applications in a mobile device [6], [7]. On the one hand, it allows the monitoring of energy consumption according to the frequency and the total number of operations, on the other hand, to put into practice our proposed methodologies through several concrete case studies.

To identify the parameters of the model, our methodology must determine the type of correlation between the number of total operations and the elementary energy dissipated at a previously defined frequency. The total number of operations is calculated by summing the active clock cycles of each processor per measurement interval. This active clock cycle number is the load of the CPU multiplied by the measurement interval duration and by the CPU frequency. The dissipated energy during a measurement interval is the product between the battery power and the measurement interval duration.

The average consumption per clock cycle can be expressed as follows [3]:

$$E_{\mathrm{clock}\;\text{cycle}}=\mathrm{Regression}\left(\sum_{i=1}^{4}{\mathrm{Load}}_{i},E_{\mathrm{dissipated}}\right)$$

*E*_clock cycle_ shows the total energy per clock cycle, Load_*i*_ the load of CPU *i* and *E*_dissipated_ corresponds to the dissipated energy measured experimentally.

Our methodology consists in establishing a regression of energy per measurement interval as a function of the total number of operations. The research presented proposes a methodology to evaluate and modeling the energy costs of mobile devices.

The proposed methodology is developed in three stages:

• data collection,
• data preparation,
• energy consumption modelling.

The aim is to monitor energy consumption by acting on the following parameters:

• frequency of the processors,
• initial level of the battery,
• energy dissipated by the clock cycle.

### 2.2 Tools data collection

The tools used to develop our methodology are [4]:

1. Trepn Profiler, a diagnostic tool for profiling performance and power consumption of Android applications. All tests of this experimentation were processed by version V6.2s. Trepn profiler provides information on system status, network status, graph performance, speed, processor frequency etc.
2. Cronoid, an automation tool that allows performing tasks on a regular basis (like KornShell). It also enables automatic task running when the status of the terminal changes. The version used is Cronoid-3.5.1.
3. CPU Frequency (V1.0.2), a tool that allows the user to change the CPU frequency setting to save energy or to achieve better performance.

The data collection stage of our methodology is based on the following steps:

• Preparation of the test platform (CPU frequency management based on the governor) in order to have the rights to fix the frequencies of the CPU with the CPU Frequency tool.
• The role of the Cronoid tool is to automate tasks in order to minimize interaction with the user.

In the case of variable frequencies, the same methodology can be applied by filtering the rows of the measurement table recorded by group of identical frequencies. To collect the data, a “Tour de France” was carried out using an electrical wheelchair to perform measurements in a diversified environment based on several scenarios. details related to the location of the measurements are detailed in Table 12

**Table 12:** Measurement locations in the scientific “Tour de France”.

Name of the city	Visit date
Lille	02 Mai 2016 (Departure)
Amiens	02 Mai 2016
Rouen	03 Mai 2016
Le Havre	04 Mai 2016
Caen	05 Mai 2016
Rennes	07 Mai 2016
Nantes	08 Mai 2016
La Rochelle	09 Mai 2016
Bordeaux	12 Mai 2016
Toulouse	15 & 16 Mai 2016
Montpellier	19 Mai 2016
Avignon	20 Mai 2016
Grenoble	23 Mai 2016
Lyon	24 Mai 2016
Dijon	26 Mai 2016
Troyes	28 Mai 2016
Paris	30 Mai 2016
Valenciennes	02 Juin 2016
Lille	03 Juin 2016 (Arrival)

The tool used for all the experiments is a tablet (HP ProSlate 8) which was previously rooted. This choice was linked to the capacity of the battery which allows a large number of tests for each charge cycle, as well as the architecture of the processor (Snapdragon 800) for its performance, minimal power use and temperature. The full characteristics of this tablet are :

• **Operating system:** Android 4.4.4 (KitKat)
• **Processor:** QUALCOMM Snapdragon 800
• **Max. CPU frequency:** 2.3 GHz
• **Number of cores:** 4
• **Sensors:** Accelerometer, ambient light, proximity, compass, barometer,gyroscope, Hall effect
• **Number of batteries:** 1
• **Technology:** Lithium polymer
• **Autonomy:** Up to 13.75 h
• **Operating temp mini:** 0^∘^C
• **Operating temp maxi:** 40^∘^C
• **Storage temp mini:** −20^∘^C
• **Storage temp maxi:** 60^∘^C

## 3 Data Exploitation

The purpose of our study is to provide detailed monitoring of the main sources of energy consumption. In the first case, only the Global Mobile Communications system (GSM) is active. The other communication networks (third generation of mobile wireless telecommunications (3G), fourth generation of mobile wireless telecommunications (4G), Wireless Fidelity (Wi-Fi) and Global Positioning System (GPS) remain inactive throughout the different experiences.

The second case aims at evaluating and measuring the energy consumed in connected use cases. Sources related to the data communications are activated completely (3G/4G or Wi-Fi) or partially (GPS) depending on the scenarios of the experiment. Another mobile device was present to serve as an access point for sharing the Wi-Fi connection.

We have developed a methodology based on the processor frequency parameters, the dissipated energy and the initial level of the battery [3]. To this end, part of the collection of the produced experimental data was analyzed by statistical tools. To validate our model, we compared our results with other existing models.

We have presented a methodology to build a model of energy consumption of applications on mobile devices. This methodology begins with recording precise measurements of energy consumption when using the applications. The strong point of our methodology is in the technique of processing recorded data which leads to a precise model. The proposed solution can be used to define an optimal frequency for one or more applications in order to provide a better experience for users with reduced energy consumption.

For the implementation of our methodology, we selected the following parameters, obtained by Trepn Profiler:

• Total load per CPU,
• Memory usage,
• CPU frequency,
• Battery level,
• Battery power.

### 3.1 Disconnected state

**Fig. 1:**
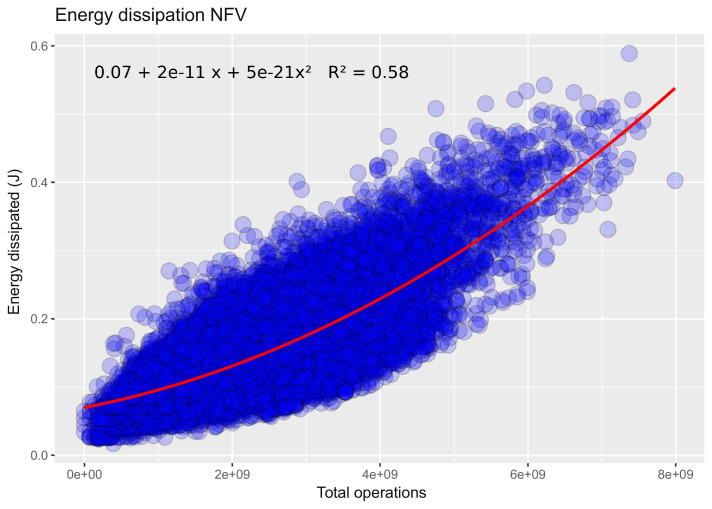
Energy dissipation for LVFF model, Frequency=1.5GHz [3].

The measurements of the dissipated energy were carried out according to the number of total operations at a fixed frequency. Fig. 1 show example of experimentation by setting the processor frequencies at 1.5 GHz.

### 3.2 Connected state

**Fig. 2:**
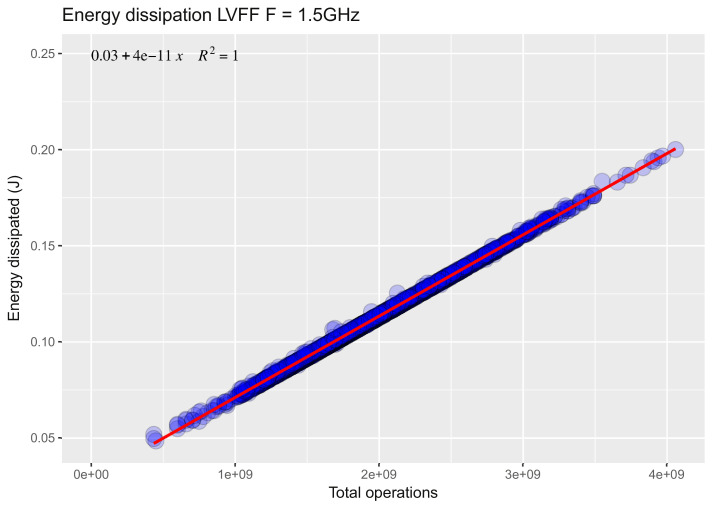
Energy dissipation for NFV scenario (default frequency) [3].

The aim of this section is to follow behaviour the energy consumption variation by activating Wi-Fi. To stay in the same context, the remote video was identical to that used in disconnected mode. We determine the type of correlation between energy consumption and the number of total operations, then we compare the obtained result with those in offline mode. Fig. 2 shows an example of experimentation for a remote video in navigation mode with variable frequency (NFV).

Raw data sources of this figure is stored in:

**NVF_default_freq_youtube_with_WiFi_Carcassonne_Narbonne.csv** at Zenodo platform: https://zenodo.org/record/3739472

In this study, we have treated the different types of dependencies between energy dissipation and the frequency of several cases in disconnected mode (LVFF, LVVF) and connected mode (NFF, NVF).

The difference between the types of regressions can be explained in part by the variability of the signal strength received for GPS and Wi-Fi (for connected mode). On the other hand, the scenarios concerned in connected mode require the activation of other parameters, which increases the rate of inputs / outputs which are partly responsible for the additional energy cost.

## Ethics Statement

Our work does not involve any use of human subjects nor animal experiments. The data are purely technological.

## Declaration of Competing Interest

The authors declare that they have no known competing financial interests or personal relationships that could have appeared to influence the work reported in this paper.

## Appendix A Supplementary Materials

Supplementary Data S1 Supplementary Raw Research Data. This is open data under the CC BY license http://creativecommons.org/licenses/by/4.0/ Supplementary Data S1
